# Effects of the Tempering and High-Pressure Torsion Temperatures on Microstructure of Ferritic/Martensitic Steel Grade 91

**DOI:** 10.3390/ma11040627

**Published:** 2018-04-19

**Authors:** Artur Ganeev, Marina Nikitina, Vil Sitdikov, Rinat Islamgaliev, Andrew Hoffman, Haiming Wen

**Affiliations:** 1 Institute of Physics of Advanced Materials, Ufa State Aviation Technical University, Ufa 450008, Russia; nik.marina.al@gmail.com (M.N.); svil@ugatu.su (V.S.); rinatis@mail.ru (R.I.); 2 Department of Mining and Nuclear Engineering, Missouri University of Science and Technology, Rolla, MO 65409, USA; ahxkw@mst.edu (A.H.); wenha@mst.edu (H.W.); 3 Department of Materials Science and Engineering, Missouri University of Science and Technology, Rolla, MO 65409, USA

**Keywords:** ferritic/martensitic steel, high-pressure torsion, ultrafine-grained structure

## Abstract

Grade 91 (9Cr-1Mo) steel was subjected to various heat treatments and then to high-pressure torsion (HPT) at different temperatures. Its microstructure was studied using transmission electron microscopy (TEM) and X-ray diffraction (XRD). Effects of the tempering temperature and the HPT temperature on the microstructural features and microhardness in the ultrafine-grained (UFG) Grade 91 steel were researched. The study of the UFG structure formation takes into account two different microstructures observed: before HPT in both samples containing martensite and in fully ferritic samples.

## 1. Introduction

Recently, much attention has been paid to research into the microstructure and properties of ferritic/martensitic steels and their use at elevate temperatures [1,2,3,4,5]. In particular, various thermal and thermomechanical treatments were applied to Grade 91 steel to study changes in microstructure [1,2]. For example, the thermomechanical treatment proposed in [5], where the steel was deformed in the austenitic regime, was aimed at refining the austenite grains and enhancing the effect of impeding dislocations by carbonitrides [3]. Warm rolling was used to increase the density of dislocations, grain boundaries, and subgrains as the nucleation sites of stable fine particles. It was demonstrated that main microstructural features which contribute to the enhancement in strength are reduced width of martensitic laths, and the presence of thermally stable precipitates [4,5]. 

At the same time, the strength of martensitic steels can be enhanced by grain refinement, using severe plastic deformation (SPD) [6,7], which is based on the application of high strains at low homologous temperatures. For example, equal-channel angular pressing (ECAP) allows the production of ultrafine-grained (UFG) structure in ferritic/martensitic steels, which increases their strength [8]. Therefore, research into the effect of grain refinement on strength of ferritic/martensitic steels are of special interest. For example, in [9,10,11], the formation of an ultrafine microstructure was attributed to the fine martensite starting microstructure. At the same time, it is recognized that the most significant grain refinement can be achieved by applying high-pressure torsion (HPT) [12]. The grain refinement to 130 nm in ferritic/martensitic steels produced by HPT was observed in [13,14]. Application of HPT to transformation induced plasticity (TRIP) steel leads to a gradual reverse transformation from body centre cubic (bcc) α’ phase to the hexagonal close-packed (hcp) ε phase [15]. A strong grain refinement at the HPT treatment was also noticed in austenitic stainless steels [16,17,18,19,20], which was accompanied by a deformation-induced martensitic transformation. However, the effect of pre-processing microstructure (purely ferrite or partially containing martensite) on microstructure and properties of UFG ferritic/martensitic steels processed by HPT has not been study. Therefore, the main focus of this work was on the investigation of the impact of pre-processing microstructure on post-processed microstructure of ferritic/martensitic steel Grade 91 subjected to HPT.

## 2. Materials and Methods 

Hot-rolled steel Grade 91 rods, whose chemical composition is displayed in Table 1, were used as starting material for investigations. The as-received rods were heated in the austenitic regime at 1050 °С for 1 h and quenched in oil. Then, the quenched samples were tempered at two different temperatures above and below the martensitic transformation temperature. It is known that the martensitic transformation temperature in steels with similar chemical composition starts from approximately 600 °С [21]. Therefore, two tempering temperatures were chosen in this work: (1) tempering at 800 °С leading to complete formation of ferrite, henceforth this treatment is referred to as T800, and (2) tempering at 500 °С where the retention of residual martensite was expected, henceforth this treatment is referred to as T500.

After the tempering, discs with the initial thickness of 1.5 mm were cut out of rods and subjected to HPT. During HPT, the shear strain γ along the radius r is given by γ = 2πrn/h where h is the thickness of the disk and n is the number of rotations. It should be noted that in recent publications it was shown that this equation is true only for a certain condition [22,23]. The samples were deformed under the pressure of 6 GPa for 10 rotations, with a rotation rate of 0.2 rpm at 20 and 300 °С. The Vickers microhardness (HV) measurements were performed with a microhardness tester from Buehler (Micromet 5101, Uzwil, Switzerland) with a load of 100 g and a dwell time of 15 s. Each measurement was carried out at a distance Rn to the disk edge. For each sample, such measurements were made by at least 6 lines. The microstructure was studied using a transmission electron microscope (TEM) JEOL JEM 2100 (JEOL Ltd., Tokyo, Japan). The samples for TEM were cut from half of radius of the HPT disc, and thinned to electron transparency by jet electropolishing in a solution of perchloric acid in butyl alcohol with a voltage of 56 V. The grain size was estimated from TEM on the bright and dark field images, where grain boundaries are clearly visible. The grain size was estimated with the grain-by-grain measurement method, using manual measurement of approximate minimum and maximum diameter of each grain. Average values were obtained from 200–300 measurements. X-ray diffraction (XRD) was performed using a DRON-4М diffractometer (Bourevestnik Inc., S-Peterburg, Russia) with CoKα radiation (35 kV, 30 mA). The reflected beam was filtered using graphite monochromator. The lattice parameter, size of coherent scattering domains (CSD), and dislocation density were estimated using the whole pattern approach, which was implemented in the PM2K software (version 2.10) [24]. The instrumental broadening of diffraction lines, i.e. the parameters U, V, W, a, b, and c for the Cagliotti function, were determined by processing a diffraction pattern from LaB6, which was obtained in the same conditions as the studied samples.

## 3. Results

**Figure 1:**
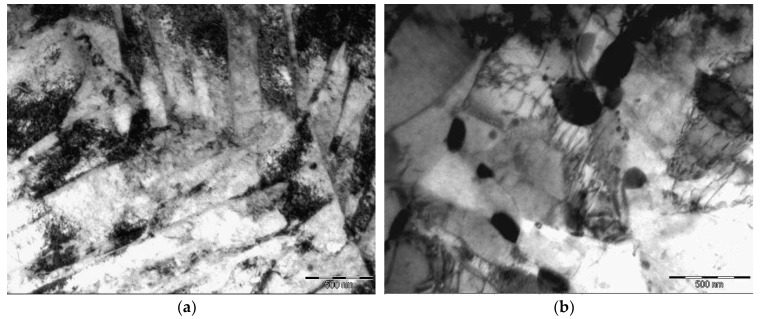
Bright-field transmission electron microscopy (TEM) images of Grade 91 microstructure after quenching and tempering at: (**a**) 500 °C; (**b**) 800 °C

Figure 1 displays TEM images showing the microstructure of the Grade 91 steel after tempering at two different temperatures. Tempering at 500 °С leads to formation of martensite laths with an average width of ~200 nm at the boundaries of prior austenite grains. After tempering at 800 °С, the average grain size is ~0.5 µm. In addition, carbide precipitates ~0.2 µm in diameter are detected. 

**Figure 2:**
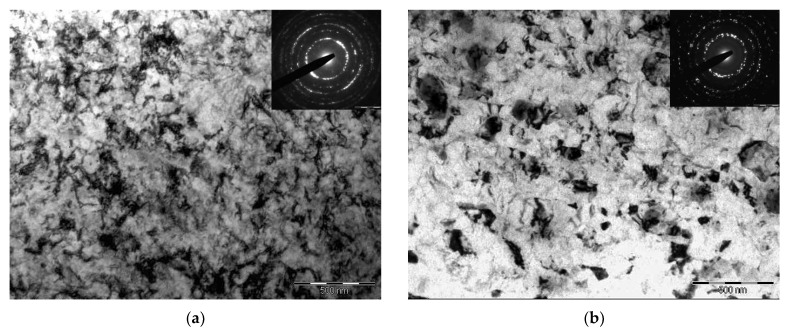

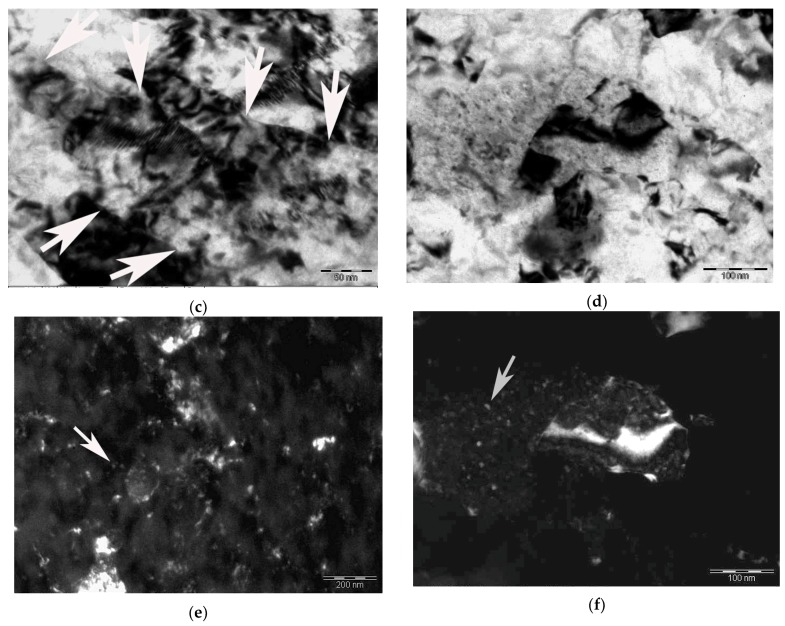
Bright-field TEM images of Grade 91 microstructure: (**a**,**c**) after tempering at 500 and HPT at 20 °С, where the arrows show the initial boundaries of martensite laths; (**b**,**d**) after tempering at 800 and HPT at 20 °С; dark-field TEM images of the Grade 91 microstructure; (**e**) TEM after T500 + HPT300 where the arrows show carbides M_23_C_6_; (**f**) after T800 + HPT200 where the arrows show MX carbonitrides.

Figure 2 shows the microstructure of Grade 91 steel after HPT. HPT at room temperature resulted in dramatic grain refinement. Grains with an average length of ~250 nm and width of ~100 nm which were stretched along the initial martensite laths, are formed in samples after T500 + HPT20 (Figure 2a). Equiaxed grains with an average diameter of ~250 nm are observed after T800 + HPT20 (Figure 2d). The formation of elongated grains along the deformation direction at lower magnification (Figure 2b) can be seen.

**Figure 3:**
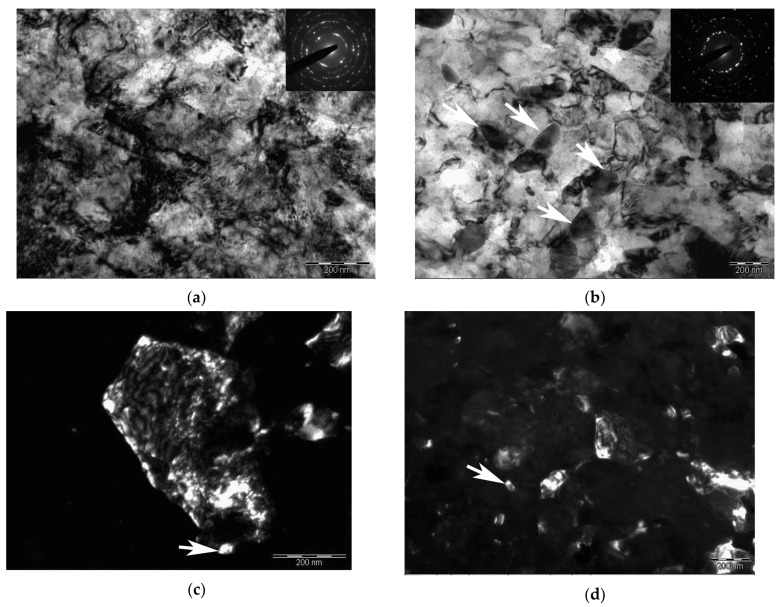
Bright-field TEM images of Grade 91 microstructure: (**a**) after T500 + HPT300; (**b**) after T800 + HPT300, where the arrows show carbides M_23_C_6_; dark-field TEM images of Grade 91 microstructure (**c**) TEM after T500 + HPT300 where the arrows show carbides M_23_C_6_; (**d**) after T800 + HPT300 where the arrows show carbonitrides.

It is known that an increase in HPT temperature activates the processes of dislocation climb and accelerates the diffusion of carbon and other alloying elements in steels. This makes it possible to reduce the density of defects in the microstructure, and achieve a more homogeneous distribution of carbides. The grain length along the martensite laths is ~450 nm, and the width is ~200 nm in steel after T500 + HPT300 treatment (Figure 3a). Equiaxed grains with an average diameter of ~350 nm are formed in steel after T800 + HPT300 (Figure 3b). 

**Figure 4:**
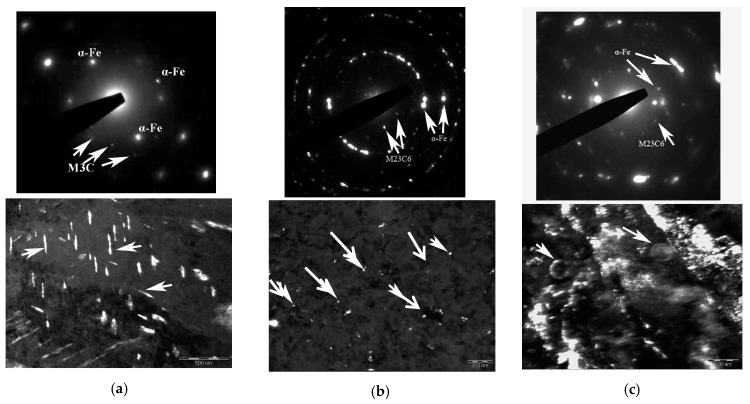
Diffraction patterns and dark-field TEM images showing carbides after various treatments: (**a**) T500, dark field in the spot (121) M_3_C; (**b**) T500 + HPT20, dark field in the spot (420) M_23_C_6_; (**c**) T500 + HPT300, dark field in the spot (420) M_23_C_6_.

Plate precipitates of carbides up to ~200 nm long were seen in the initial samples after T500 treatment (Figure 4a). After T500 + HPT20, additional precipitation of carbides with an average diameter of ~10–30 nm was observed too, Figure 4b. The increase in the HPT temperature at T500 + HPT300 treatment resulted in some spheroidization of carbides and an increase in their sizes up to ~50 nm, Figure 4c.

**Figure 5:**
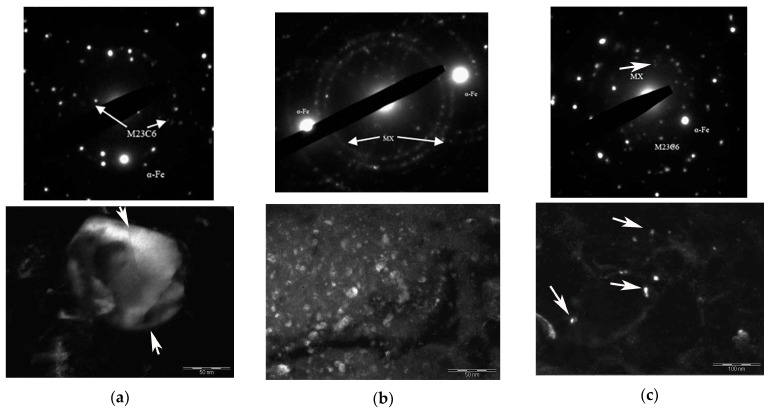
Diffraction patterns and dark-field TEM images showing particles after various treatments: (**a**) cutting of carbides by dislocations in steel subjected to T800 + HPT20; (**b**) МХ carbonitrides after T800 + HPT20, dark field in the spot (111); (**c**) МХ carbonitrides after T800 + HPT300 dark field in the spot (200).

After the T800 treatment, coarse particles of carbides of ~100–300 nm were observed in the microstructure. The carbides were located mainly at grain boundaries, and dispersed particles of fine secondary MX (М—V, Nb, Ti, Mo; Х—С, N) carbonitrides with an average size of ~50 nm were found at grain boundaries and at prior austenite boundaries (Figure 3b). The T800 + HPT20 treatment resulted in additional precipitation of carbonitrides from the solid solution, and the particles with sizes of ~5–10 nm precipitated mainly inside grains (Figure 5b). The sizes of coarse carbides were not changed during HPT, and other carbides with sizes <100 nm were probably cut by dislocations (Figure 5a). It is known that the smaller particles can be easily cut by dislocations, in comparison with coarse particles [25]. The increase in the HPT temperature to 300 °С at the T800 + HPT300 treatment resulted in the same sizes of carbides ~100–300 nm.

**Figure 6:**
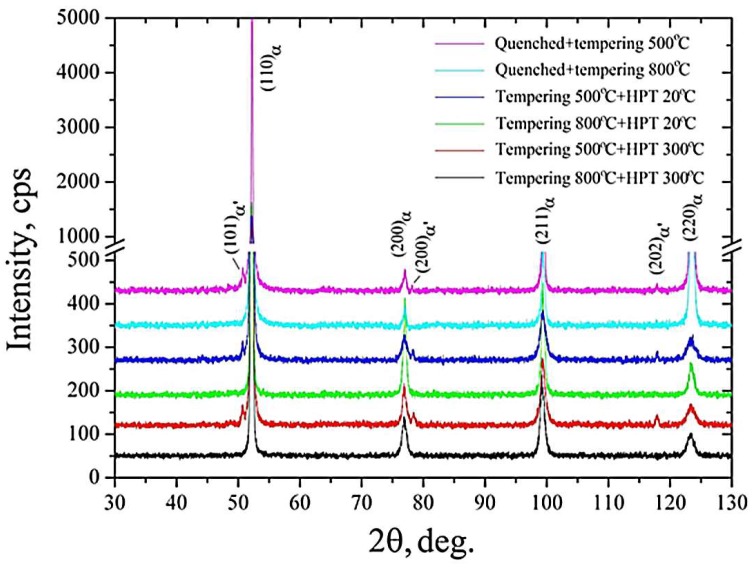
X-ray diffraction patterns of the Grade 91 steel after various treatments.

Figure 6 displays the X-ray diffraction patterns of the samples subjected to various treatments. In all samples studied, the X-ray diffraction patterns are characterized by the set of intensive maxima with Miller indexes (110), (200), (211), and (220) that correspond to the α-Fe phase. After tempering at 500 °С, the martensite phase with the volume fraction of 6.6% was noticed, whereas after tempering at 800 °С, this phase was not observed. The HPT processing of the samples after tempering at 500 °С resulted in further increase in the volume fraction of the martensite phase. In particular, after T500 + HPT20, the volume fraction of the martensite phase was 8.3%, and after T500 + HPT300, it was 10.2%.

The X-ray diffraction studies showed that the increase in HPT temperature up to 300 °С led to reduction in the lattice parameter. In particular, the most significant reduction in the lattice parameter was seen in the samples subjected to the T800 + HPT300 treatment. The lattice parameter value after this treatment (Table 2) is significantly higher compared to that typical of pure ferrite (0.28660 nm [26]). Additionally, it is smaller in comparison with the value for other researched samples. The tendency for the reduction in the lattice parameter in the sample after T800 + HPT300 may suggest that after HPT processing at 300 °С, the ferrite matrix is depleted in solutes, and precipitates with a higher volume fraction are formed. Precipitates were observed during the TEM studies.

Additionally, the increase in HPT temperature from 20 °C to 300 °C results in an increase in CSD size by more than 1.5 times and decrease in dislocation density by more than two times, independent of tempering temperature (Table 2). 

**Figure 7:**
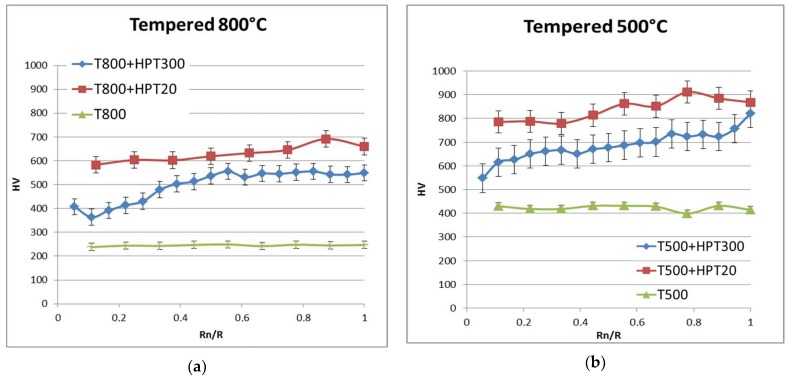
Vickers microhardness plotted against the distance to the center in the HPT samples: (**a**) red—T800 + HPT20, blue—T800 + HPT300, green—tempered sample; (**b**) red—T500 + HPT20, blue—T500 + HPT300, green—tempered sample. Rn/R—distance from center of the disc.

Figure 7 displays graphs of the microhardness values as a function of the distance to the center of the samples. The average microhardness of the sample after the T800 treatment is 250 HV and increases up to 650 HV after the T800 + HPT20 treatment. The difference between the minimum value in the disc center and the maximum value at the edge, does not exceed 100 HV. The increase in the HPT temperature to 300 °С leads to a gradient in microhardness along the sample radius up to 200 HV. At the same time, the average microhardness decreases to 490 HV. The microhardness after T500 treatment is 420 HV. After the additional HPT20 the average microhardness achieves 840 HV. The inhomogeneity along the radius remains at the level of 100 HV. The increase in the HPT temperature to 300 °С reduces the average value of microhardness to 630 HV. The heterogeneity along the sample radius increases up to 200 HV. 

## 4. Discussion

It is well known that martensite is an oversaturated solid solution of carbon in α-Fe and is characterized by a complex structure [27]. The high strength of martensite is due to several hardening mechanisms: solid solution, dislocations and grain boundaries. Short-term tempering is required to reduce internal stresses prior to deformation of the martensitic steel by HPT [28]. The T500 + HPT20 treatment resulted in striking grain refinement in the structure. There are high dislocation densities in the samples owing to the imposed high plastic strain (Table 2). It is known that dislocations play a significant role in the process of carbides dissolution in martensitic steels during deformation [29]. Such dislocation density in Grade 91 steel induces the formation of the metastable carbides in tempered samples (Figure 4a) and leads to the formation of nanoparticles after T500 + HPT20 (Figure 4b). 

The samples after T500 + HPT20 treatment have the average microhardness of 8.4 GPa, which is two times higher than microhardness in the tempered samples. High microhardness is evidently caused by dramatic grain refinement (Figure 3), dispersion hardening (Figure 4), and high dislocation density (Table 2). During HPT processing at 300 °С, the diffusion process was enhanced in UFG steel. Formation of UFG structure during T500 + HPT300 is influenced by the two known processes: grain refinement by severe plastic deformation, and dynamic recovery in the martensite structure [23,24,25,26,27,28,29,30,31,32]. The increase in HPT temperature to 300 °С led to reduced dislocation density (Table 2) and increased grain size, compared to samples processed by HPT at 20 °C. The observed carbides had a globular shape and grew to 50 nm. In this case, the average microhardness after HPT300 is reduced by 25% compared to HPT20, due to multiple factors, including increased grain size and reduced dislocation density. 

The dislocation density in the samples processed with T800 + HPT300 is reduced three times to 3.5 × 10^−15^ m^−2^, compared to the samples after T800 + HPT20. The average grain size of the samples after T800 + HPT300 is ~350 nm, and reduction in the sizes of coarse carbides to 200 nm is observed. The size of carbonitrides for T800 + HPT300 does not change compared to that of T800, and their redistribution at grain boundaries takes place. This suggests that deformation-induced migration of grain boundaries in HPT samples may be hindered by carbonitrides which are located at grain boundaries. Such distribution of particles is favorable for achieving enhanced creep resistance at temperatures up to 650 °С. 

## 5. Conclusions

The grain refinement, microstructural evolution, and precipitation of secondary phases in the Grade 91 steel subjected by HPT were studied using transmission electron microscopy (TEM) and XRD methods. It was shown that the initial microstructure and deformation temperature is important for grain refinement and forming the properties of steel under study. The main results can be summarized as follows:

1. After HPT of the samples containing martensite (tempered at 500 °С before HPT), the microhardness increases by two times to 8.4 GPa. Such microhardness is attributed to several factors: localization of plastic deformation inside martensitic plates inducing the formation of grains of 100–200 nm in size, the high degree of solid solution saturation, and high dislocation density. This may be due to the fact that the alloying elements necessary for the precipitations of carbonitrides remained in the solid solution.
2. The increase in the microhardness after HPT processing of the ferritic samples (tempered at 800 °С before HPT) occurs mainly due to grain boundary strengthening. The microhardness increases by three times up to 6.4 GPa compared to the samples before HPT.
3. The increase in HPT processing temperature from room temperature to 300 °С results in enhanced carbon diffusion, recovery, and significant reduction in the dislocation density.
4. Severe plastic deformation has practically no significant effect on the refinement of coarse M_23_C_6_ carbides. Their fragmentation is possible if the carbide size does not exceed 100 nm. The increase in the temperature of the deformation leads only to a smaller scatter in size.

## Figures and Tables

**Table 1:** Chemical composition of steel Grade 91, wt %.

С	Mn	P	S	Cu	Si	Ni	Cr	Mo	V	Ti	N
0.08	0.53	0.016	0.003	0.09	0.28	0.13	8.43	0.9	0.225	0.01	0.038

**Table 2:** Microstructural parameters from X-ray diffraction.

State	Lattice Parameter (nm)	Size of CSD (nm)	Dislocation Density (10^−15^ m^−2^)
500C +HPT 20C	0.287957(2)	34(3)	22.4(2)
500C + HPT 300C	0.287869(11)	57(4)	11.9(3)
800C + HPT 20C	0.287802(10)	36(3)	10.5(4)
800C + HPT 300C	0.287436(7)	61(4)	3.5(2)
